# Ten quick tips for homology modeling of high-resolution protein 3D structures

**DOI:** 10.1371/journal.pcbi.1007449

**Published:** 2020-04-02

**Authors:** Yazan Haddad, Vojtech Adam, Zbynek Heger

**Affiliations:** 1 Department of Chemistry and Biochemistry, Mendel University in Brno, Brno, Czech Republic; 2 Central European Institute of Technology, Brno University of Technology, Brno, Czech Republic; University of Toronto, CANADA

## Abstract

The purpose of this quick guide is to help new modelers who have little or no background in comparative modeling yet are keen to produce high-resolution protein 3D structures for their study by following systematic good modeling practices, using affordable personal computers or online computational resources. Through the available experimental 3D-structure repositories, the modeler should be able to access and use the atomic coordinates for building homology models. We also aim to provide the modeler with a rationale behind making a simple list of atomic coordinates suitable for computational analysis abiding to principles of physics (e.g., molecular mechanics). Keeping that objective in mind, these quick tips cover the process of homology modeling and some postmodeling computations such as molecular docking and molecular dynamics (MD). A brief section was left for modeling nonprotein molecules, and a short case study of homology modeling is discussed.

## Introduction

Protein 3D-structure folding from a simple sequence of amino acids was seen as a very difficult problem in the past. However, it has progressed through the years into an operable challenge with amenable and reasonably accurate predictions in many cases [1,2]. According to the funnel hypothesis of the protein potential energy landscape, the native-protein conformation (3D structure) is at the bottom of the funnel at the lowest free energy, i.e., a global energy minimum [1]. A variety of computational strategies have been developed to face the challenges in determining the native conformations of proteins by exploring (scanning) the potential energy of the conformational space (c-space) [3]. These strategies are divided into either deterministic or heuristic algorithms, differing in the search coverage of the c-space [3]. Briefly, a deterministic approach scans the entire or part of the c-space, mostly by exclusion of subspaces based on a priori knowledge, e.g., homology modeling allows experts to predict protein 3D structure by modifying a homologous structure, thus eliminating a huge amount of c-space. A heuristic approach scans only a fraction of the c-space yet with a representative set of conformations (e.g., MD applies energy functions to study forces, solves the equations of motion, and predicts atomic trajectories in time-dependent fashion). MD provides information about the folding and unfolding pathways despite the limited c-space coverage. These strategies and others—individually, combined, or sequentially—were successfully applied for understanding of the function of macromolecules in the cell and also used for the development of industrial enzymes and pharmaceutical drugs (more algorithms, methods, and applications are reviewed in [4]).

High-resolution protein 3D structures generated by in silico prediction methods can significantly reduce the labor, time, and cost of wet-lab experiments. The gap between the number of protein sequences and experimentally determined protein 3D structures is widening. A recent estimate of the number of discovered protein sequences was shown to be 736 times larger than the number of resolved protein 3D structures compared to previous estimate of 120 times in 2006 [5].

**Fig 1:**
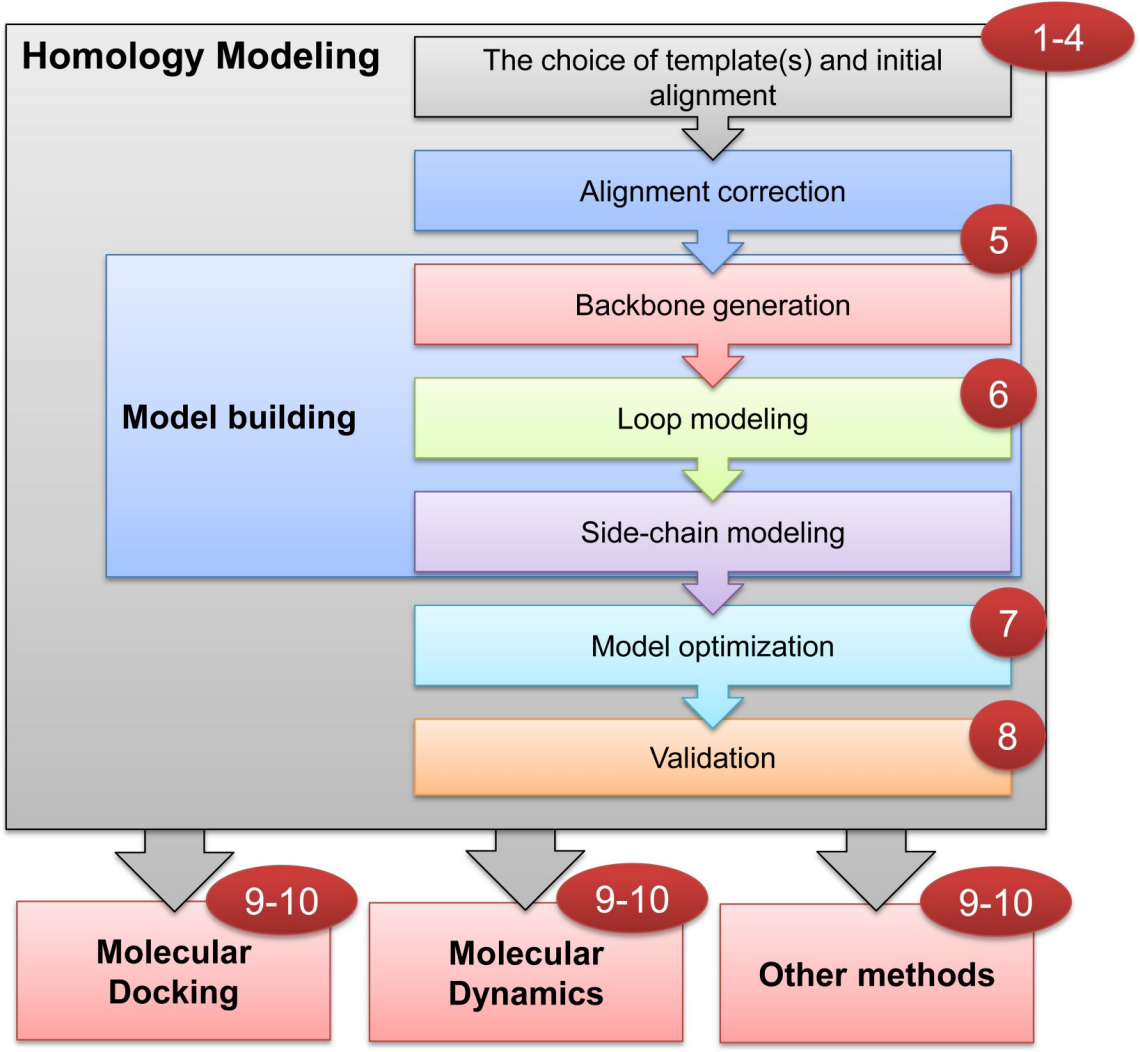
Homology-modeling workflow. Numbers show the steps where tips are applicable.

In the absence of experimentally determined protein 3D structures, homology modeling plays a cost-effective role in structure-based applications and the characterization of protein properties and functions [6]. The homology-modeling work flow is divided into seven main steps (Fig 1) [7]. The process begins by choosing the best template 3D structure, on which the target sequence can be successfully threaded. The first alignment for template search is commonly performed using BLOcks SUbstitution Matrix (e.g., BLOSUM62) [8]. A second alignment (also known as alignment correction) is used to build the backbone 3D structure. Here, the sequence and structure or multiple alignment apply a position-specific scoring matrix (PSSM) [9] or hidden Markov model (HMM) [10]. The alignment procedures are discussed in detail in Tip 5. In line with the work by Daga and colleagues [11], we recommend a comprehensive overview of alignment methods used in choosing the template and generating the backbone 3D structure and other steps crucial for successful homology modeling. A loop-modeling approach is used for correcting the folding of low-homology regions, with high accuracy for up to 12 to 13 residues [12]. Next, the side chains are reconstructed through conformational search [13] using a backbone-dependent rotamers library [14,15]. A stand-alone software called SCWRL is among the commonly used side-chain conformation prediction tools [16]. The structure should next be refined and validated by various quality-assessment tools. Five categories of sources of potential inaccuracies are observed in homology models [17]: (1) Inappropriate template selection, (2) misalignment errors, which can be reduced by using multiple alignment approaches, (3) shift errors in almost correctly aligned regions, often caused by template structure misalignment that can be compensated by using multiple template structures, (4) side-chain packing inaccuracies resulting from divergence between a given sequence and homologous template 3D structure or even divergence of identical side-chain rotamers, and (5) low-homology regions or lack of a suitable homologous 3D template, which could be quite often addressed by loop modeling.

The Critical Assessment of protein Structure Prediction (CASP; predictioncenter.org) initiative is an experiment performed every two years since 1994. The CASP has shown continuous interest from researchers and continuous improvement in accuracy of protein 3D-structure prediction. In the latest experiments (CASP12 and CASP13), homology modeling advanced in four major areas: Sequence–structure alignment, combining 3D-structure templates, loop modeling, and protein assembly [18,19]. Modelers are encouraged to follow the latest CASP results. Significant improvements in homology-modeling accuracy were shown in the latest CASP competitions, attributed mainly to use of multiple templates, ab initio modeling of missing parts, improved refinement, and enhanced estimation of model accuracy [20]. The Yang Zhang group, who won the CASP13 competition (positions 1, 3, and 5) and several previous CASPs, developed three automated servers (zhanglab.ccmb.med.umich.edu)—namely, “Zhang” and “Zhang‐Server” using Iterative Threading ASSEmbly Refinement (I-TASSER) and “QUARK,” which was enhanced by incorporation of a sequence‐based contact prediction producing contact maps for soft restraining, thus improving quality of predicted models [21,22].

The Continuous Automated Model EvaluatiOn (CAMEO; www.cameo3d.org) project provides weekly follow up for three modeling servers groups: (1) Homology modeling, (2) model quality estimation, and (3) contact prediction. At the time of writing, there were 16 homology-modeling servers registered. To name a few of the commonly used servers: SWISS-MODEL (swissmodel.expasy.org) [23], Robetta (www.robetta.org) [24], Protein Homology/AnalogY Recognition Engine 2 (Phyre2; www.sbg.bio.ic.ac.uk/phyre2/) [25], RaptorX (raptorx.uchicago.edu) [26], and Position Specific Iterated-BLAST–based secondary structure PREDiction (PSIPRED; bioinf.cs.ucl.ac.uk/psipred/) [27]. On the other hand, many public and commercial programs are available and widely used, notably MODELLER [28], developed by the Andrej Sali group; SCWRL and MolIDE [16], developed by the Roland Dunbrack group; and Prime [29], affiliated with Schrödinger software.

## Tip 1: The choice of the template(s) is a deal breaker

The Worldwide Protein Data Bank (wwPDB) maintains the largest, public, free archive of macromolecular structural data (www.wwpdb.org). The latest 3D-structure file format is the Protein Data Bank (PDB) macromolecular Crystallographic Information File (PDBx/mmCIF) format, which substituted the legacy PDB format and has recently become the only acceptable format for deposition in the wwPDB [30]. The PDBx/mmCIF format is robust for programming applications as well as for supporting more relevant chemical, experimental, and complex data types [31]. There are several criteria to take into consideration when choosing the template(s) for homology modeling from the database. X-ray crystallography and nuclear magnetic resonance (NMR) resolved structures vary in resolution; however, cryo-electron–microscopy 3D-structure resolution is too low for the homology-modeling strategies described here. Initially, the modeler performs a protein Basic Local Alignment Search Tool (BLAST) search (blast.ncbi.nlm.nih.gov) using target protein sequence as the query and PDB as database. BLASTp (p stands for protein) search is sufficient to know which 3D-structures have high identity and coverage of the query. The degree of homology is one factor to consider since identities (i.e., the number of identical amino acids in a sequence alignment) below 25% are difficult to model [7]. In the case of deletions and/or insertions or shifts in the alignment, a Position-Specific Iterated BLAST (PSI-BLAST) (discussed in Tip 5) could be more successful. As mentioned earlier, the combination of multiple homologous 3D templates usually improves the accuracy of modeling. Multiple or alternative templates can be already present within the same PDBx/mmCIF under different chain or model names or as monomer parts of an oligo-protein. BLAST results show the chain name after the PDB accession (PDB and PDBx/mmCIF accessions consist of four characters). The size of the protein can influence the results of homology modeling. This is particularly obvious when validation is done by a knowledge-based scoring method (discussed in Tip 8). A decision to avoid some protein domains (either absent in template or badly modeled) can be justified if there is no direct or clear functional role of them. If a highly nonconserved region is short (i.e., no longer than 13 residues), it can be modeled as the loop discussed in Tip 6, otherwise an ab initio approach is recommended.

## Tip 2: Read the article that published the homologous template 3D structure

This seems a bit unfair for researchers downloading hundreds of 3D structures from the database for large-scale analyses. However, with a high-resolution model in mind, a PDBx/mmCIF format containing atomic coordinates alone can be sometimes misleading. The modeler should understand the experimental procedures used to produce the crystal structure and explore the previous literature history. The history of the 3D structure gives the modeler a thorough justification for using it and predicts its implications, keeping in mind that a bad template is likely to yield a bad model. The article that published the 3D structure provides insights to the biological mechanisms involved and protein domains’ functions. Furthermore, it is important to be familiar with established annotations, domain names, and special nomenclature for that particular structure in literature.

## Tip 3: Remove unnecessary elements, solvents, ligands, and ions

The legacy PDB file format is a readable text file organized in records (one record for each atom). The PDBx/mmCIF format added customizable categories in order to tailor the records for more applications. Details of formatting can be found in the PDBx/mmCIF dictionary resources (mmcif.wwpdb.org). A standard residue (amino acid or nucleic acid) consists of several ATOM records. A chain of residues (from amino to carboxyl terminus) is terminated by TER record while the nonstandard residues are described in HETATM records (including water atoms). The orthogonal xyz coordinates are described in angstroms (Å). Horizontally, each record is 80 characters long. Atomic coordinates’ data are divided into specified lengths and data types (e.g., string or integer). Columns 1 through 6 contain the record name. Columns 7 through 11 contain the atom serial number and so on. The majority of software has limitations for dealing with nonstandard record types. Unless the solvents (e.g., water molecules) or ions are integral part of the interaction interface, they must be removed prior to molecular docking or MD simulation to alleviate errors. In fact, a careful text cleanup of a PDB file retaining only ATOM and TER records (and some HETATM records) can simplify and facilitate identification of errors in further steps of modeling. Format conversions between PDB and mmCIF and consistency checks can be performed via RCSB MAXIT suite (sw-tools.rcsb.org/apps/MAXIT/) [32].

## Tip 4: Refine the 3D structure and introduce hydrogens (if missing)

Coming across missing atoms is very common in crystal structures since the researchers try to preserve only the most high-resolution elements. This includes parts of the 3D structure that are difficult to detect with certainty. In addition, the 3D structure might be derived from a rather more-stable mutant form. Variations in residue numbering that deviate from the numeration of the protein sequence in the Uniprot database (www.uniprot.org) are important and can pop up even at the stage of making final figures. Some software are sensitive to naming of chains, to gaps in numbering, and to nonstandard nomenclature of atoms or residue. Almost all protein crystal structures saved in PDB format lack hydrogens due to low resolution in crystal. It is important to add the hydrogens to the 3D structures before performing docking or simulations, and such process can be done in most software packages. However, there is more to this task than clicking on a “add hydrogen” command, as will be discussed in Tip 9. The addition of hydrogen should fill all the missing valences, minimize atomic clashes, and facilitate hydrogen bonding in the protein structure. The Reduce program developed by the David and Jane Richardson group adds hydrogens for proteins and nucleic acids in standardized geometry with optimization of the orientations of the functional groups [33]. Accurate addition of hydrogens plays a role in 3D-structure refinement and in analyzing hydrogen bonding. The Hydrogen Atom ADdition (HAAD) algorithm was developed by the Yang Zhang group by applying basic rules of orbital hybridization followed by optimization of steric repulsion and electrostatic interactions. HAAD has better accuracy and performance than Reduce [34]. Note that some programs only add polar hydrogens (explained in Tip 10).

## Tip 5: Mind the gaps in the alignment

We have mentioned two alignment steps in the homology-modeling process (Fig 1). The first alignment is used for searching for the template(s) and can be a simple sequence-to-sequence alignment or the BLASTp search. The second alignment step, often described as alignment correction, is the one that is actually used for generating the backbone. Before discussing alignment correction in details, it is important to know some terminology related to the alignment. For a simple pairwise alignment of two sequences, the residue mismatches (substitutions) and gaps (insertions and deletions) are scored using a matrix (higher score is better). Substitution matrices, such as the point accepted mutation (e.g., PAM250) [35] or BLOSUM (e.g., BLOSUM62) [8], give a positive or negative cumulative score based on the general evolutionary probability of amino acid mutation or based on all possible substitutions of one amino acid for another in a particular group of proteins (blocks of 62% identity for BLOSUM62), respectively. A score also has costs (penalties) for gap opening (existence) and gap extension. At the time of writing, the default setting for BLASTp is the BLOSUM62 with gap costs of 11 for opening and one for gap extension. A statistical parameter called expectation value (E-value) tells the likelihood of similarity to occur by chance (lower E-value is better).

A sequence-to-structure alignment will provide better understanding to the 3D-structure similarity. Here, the target sequence is query, and the template structure(s) is subject for position-specific or sequence-to-structure alignment done by either PSSM [9] or profile HMM [10]. In PSSM alignments such as PSI-BLAST, the position of amino acid in the sequence is weighted in the calculated matrix. This method performs well for finding homologs in variable short regions of constant lengths, and since gaps are not applicable in PSSM, they are ignored. The attempts to generalize a PSSM method for including gaps were possible through the concepts of profile HMM. In profile HMM such as HMMER (hmmer.org), the possibilities of matches, deletions, and insertions in each sequence position are represented in HMM states. Briefly, HMM is described as a linear-state machine with a series of nodes corresponding to the sequence positions. Each node can exist in one state (such as matches, deletions, or insertions). A dynamic programing algorithm is used to find the most probable path by calculating transition probability from one node to another. Finally, the “profile” will be the consensus sequence of the alignment. Profile HMMs require more computations and experience, but they are more useful with low-homology cases since it is possible to detect gaps in the alignment. As a general rule, it is recommended to use profile HMMs with identities below 35% (also where alignment gaps are obvious) while PSSM alignments and sequence-to-sequence alignments can be tolerated with identities above 35% [36]. We believe that it is very important to detect alignment gaps or “shifts” in homology modeling early [37]. The errors in sequence alignment will be inherited in the backbone, which later get amplified at the level of side chains. Once you identify the gap region(s), it is time to remodel it more accurately, which is the topic of the next tip.

## Tip 6: Consider the size and residue composition of loops during modeling

The insertions and deletions are less observed in regions of α-helix or β-sheet [38]; therefore they are often found in more-variable and less-conserved loops. Loops are important for functional specificity and can contribute to active and binding sites [39]. The loop modeling, similarly to the whole-protein modeling, requires extensive sampling of the c-space and final validation of the quality of the 3D structure [40]. Decision on how to perform loop modeling depends on the loop size and amino acid compositions, which directly affect the scanning of c-space. Regarding composition, the most critical residue variations are usually from any residue into proline and from glycine into any residue. In both cases, the new residue must fit into a more restricted range of backbone dihedral angles [7]. There are three main strategies for the loop modeling:

1. Ab initio: This method uses exhaustive search for identifying the conformation of the minimal energy through optimization algorithms using a knowledge-based scoring function. The accuracy of an algorithm is tested over different decoys. Enumeration methods are a class of ab initio methods that use a virtual database of short oligopeptide conformations. The biggest challenge for ab initio is the exponential explosion of c-space by extending the loop with each new residue [12]. The ModLoop program provided in MODELLER is one of the most commonly used ab initio methods [41]. There are also several loop-modeling methods developed for various applications, provided by the Rosetta framework [42].
2. Knowledge-based: This type of method screens the X-ray crystallography databases to find homologous conformations for a given loop sequence. ProMod3 [43] in the SwissModel server, FREAD [44], and Data-based approaching using Remote or Unrelated Structures for Loop modeling (DaReUS-Loop) [45] are good examples of commonly used knowledge-based algorithms.
3. Combined strategies: Liang and colleagues [46] combined Optimized Side-Chain Atomic eneRgy (OSCAR)-loop (i.e., *ab initio* method) and Spanner (i.e., knowledge-based potential) to achieve higher accuracy. The sampling from different initial methods followed by overall scoring is recommended for long loops (more than 10 residues).

## Tip 7: 3D-structure minimization is imperative prior to any computational analysis

It is accepted that the crystal structure is the experimental description of molecular nature. However, to apply physics laws in computational 3D modeling—ensuring accurate atomic masses, charges and bonds—researchers use a force field (FF; a set of standard parameters and equations that describe the bond lengths, bond angles, dihedral angles, improper planes, and electrostatic and van der Waals forces). FFs are discussed more in Tip 10. The minimization of FF-free energy adjusts the 3D structure to the used FF, lowers the physical errors, and ensures the reproducibility of the work by other researchers. Mathematically speaking, the process of minimizing 3D structure is performed by geometric optimization of the potential energy surface applied by multivariable algorithms to achieve convergence [47]. In simpler words, the algorithms use calculus to know if the changing variables are directing the slope of a curve downward. The algorithm may use first, second, or both derivatives to screen for descent direction. Both steepest descent (SD) and conjugate gradient (CG) methods are gradient-based iterative methods that use first derivative values (slope) of the objective functions in the algorithm to find the minimum [47]. A SD method is the simplest algorithm, yet it has relatively poor convergence and would often cycle around the minimum while rarely reaching it. It can be mostly applied to relax atomic clashes before an MD simulation or as a first step in a more complex minimization strategy. On the other hand, a CG method keeps a memory of its previous gradients through a weighted average of the gradient and the direction taken in previous iteration (thus compensating for missing information) and has much better convergence than a SD method. To solve the problem of accumulated errors in memory, the minimization process can be restarted, and the weight factor is set to zero. The Newton–Raphson (NR) methods use first and second derivative in their algorithms and show comparable results to CG methods. A good minimization protocol is to combine two or more algorithms; for example, you first use SD then follow it by CG or NR [48,49].

The convergence of the potential energy surface occurs by four criteria and used cutoff values: (1) Forces must be zero, (2) root-mean-square of forces is zero, (3) the displacement for the next step of optimization is less than cutoff value, or (4) the root-mean-square of displacement for the next step of optimization is less than cutoff value [47]. The calculation will also stop by reaching the last step of iteration. Almost all MD programs and most molecular modeling and graphic-visualization software has integrated packages for minimization. In MD programs, such as Assisted Model Building with Energy Refinement (AMBER) or GROningen MAchine for Chemical Simulations (GROMACS), the minimization parameters are specified in the input files: method of minimization, number of iterations, the cutoff criteria and their values. In graphic-visualization software such as University of California, San Francisco (UCSF) Chimera [50], DeepView/Swiss-PdbViewer [23], Pymol [51], or visual molecular dynamics (VMD) [52], these parameters are defined in window and plug-in settings. It is also possible and highly recommended to select parts of the protein that can be either fixed or minimized (see the case study section). However, programs should be able to minimize all additional nonprotein compounds, providing the availability of their topologies in the FF.

## Tip 8: Use multiple strategies for 3D structure validation and evaluation

The majority of homology-modeling programs generate a large number of protein 3D models and rank them according to various methods of scoring. Since each method of evaluation studies the 3D structure from different perspective, combining several methods could allow for more reliable evaluation. The evaluation is not necessarily the end step in homology modeling since some identified errors might require the repetition of earlier steps of the process. The evaluation methods can be divided into four groups:

1. Physics-based methods: Most of these methods are based on calculations of FF parameters and optimal stereochemistry. Casually, an evaluation for a protein X-ray crystal structure can be performed using MolProbity [53], which validates the quality from global (whole protein) and local (small regions) perspectives. The method identifies backbone outliers, side-chain outliers (rotamer deviations), and inappropriate all-atom contacts (atomic clashes). The backbone outliers (Ramachandran and Cβ deviations) are most important since they can be amplified into larger errors. Ramachandran outliers describe the deviations in Φ and Ψ dihedral angles, which correspond to the protein secondary structure. The Cβ outliers describe the deviations in the position of the Cβ atom (connected to the Cα in the backbone). Other commonly used programs include WHAT IF [54] and PROCHECK [55]. The latter program has already been added to the RCSB MAXIT suite for the purposes of checkup prior to data deposition in the wwPDB.
2. Knowledge-based methods: A database-dependent validation approach uses scores representing energies obtained statistically within the context of all known experimental 3D structures in the database. The Swiss-MODEL uses the Qualitative Model Energy ANalysis (QMEAN) to score Cβ-interaction energy, all-atoms pairwise energy, torsion angle energy, and solvation energy [56]. The MODELLER program uses two knowledge-based methods (i.e., Discrete Optimized Protein Energy (DOPE) [57] and PROtein Structure Analysis II (PROSAII) [58]) and one physics-based method (PROCHECK [55]) for evaluation. One alternative approach is the Quality Assessment (QA)-RecombineIt server, allowing users to model protein 3D structures based on consensus identifying highly-conserved regions in a wide range of input protein 3D models [59]. Here, the quality is checked by several methods either for a single model or by the clustering of multiple models. Among these methods, meta-methods for quality assessment of protein models (MetaMQAP) uses outputs from at least eight quality predictor methods to assess global and/or local accuracy of protein 3D structures [60].
3. **Machine learning-based methods:** Eramian et al. developed a composite score for predicting errors in homologous models using the support vector machine (SVM) regression method [61]. SVM is a supervised-learning algorithm that derives features from a training data set and tests them on a separate data set, which can be useful for regression, classification, or clustering. The researchers integrated 24 individual scores from different methods, combined them, and tested nearly 85,000 composite scoring functions. The most accurate score was based on a combination of four knowledge-based scores and two secondary-structure prediction scores. The former four statistical potential scores were (1) DOPE for nonhydrogen atom, (2) MODPIPE [62] for surface, (3) MODPIPE for contact, and (4) MODPIPE combined contact/surface statistical potentials. The latter two secondary-structure prediction scores were derived; first by calculating secondary-structure assignments via the Dictionary of Secondary Structure of Proteins (DSSP) method [63] then by reducing the assignments from eight to three states. Protein secondary-structure prediction based on PSSM (PSIPRED) [64] scores were then compared by percentage of amino acids having different three states. The subsequent score was also weighted by PSIPRED prediction confidence, thus resulting in two PSIPRED percent and weight scores.
4. **Experimental-based methods:** An experimental validation (with reservations to resolution) is the ultimate test for a theoretical model. All experimental data ranging from ligand binding to spectroscopy or X-ray crystallography can be used for evaluation. The simplest method for evaluation of 3D homology structure within its experimental counterpart is the root-mean-square deviation (RMSD), which gives an average for the distances between all the atoms in two 3D structures. Since minimal perturbations in a loop between domains can result in a misleading high RMSD, the method is better applied by first dividing the protein into fragments [37]. A more systematic and accurate method was developed by Adam Zemla to consider local (smaller) regions and perform local and global structure superpositions. This method is called the global distance test (GDT), and it is implemented in the CASP experiments [65]. A detailed evaluation for agreement between the 3D model and a reference (e.g., the template 3D structure) can be performed using SphereGrinder (spheregrinder.cs.put.poznan.pl/) [66]. SphereGrinder performs RMSD-based tests in localized motifs based on selected atoms in the 3D model. The localized motifs are represented by spheres of radiuses defined by users and according to different levels of quality.

It is possible to use the previous methods to manually identify and correct errors via graphical visualization software (by making a checklist and using tools to modify dihedral angles and changing rotamers). For side chains, most aromatic and long chains have similar rotamers across homologous 3D structures (unless they are facing the solution). Thus, a superposition with the template can highlight any unwanted changes. Transitions from aromatic to aliphatic side chains or from small to long side chains are most critical e.g., Phe from/to Val are common codon transitions [37]. However, some homology-modeling programs have their own refinement subprograms such as RosettaCM [67] in Rosetta, and the subprogram in I-TASSER [68]. In the case study, we perform a manual refinement to show how tricky this step can be in affecting both physics-based and knowledge-based evaluation scores.

The refinement step often overlaps with the sampling procedure that is the end goal of homology modeling, i.e., identifying the native conformation with minimal potential energy. Refinement methods and servers have been recently reviewed in [69]. Refinement pipelines consist of sampling and scoring steps; however, finding the most native conformation is challenging due to the very small variation between generated 3D structures. Among the top performers on CASP13 was the Protein structure REFinement via Molecular Dynamics (PREFMD) server (feig.bch.msu.edu/prefmd), which applies MD and structural averaging for refinement [70].

## Tip 9: Use a checklist of amino acid protonation states to mimic role of pH

The protonation of the side chain plays role in most of the hydrogen-donor and/or hydrogen-acceptor and electrostatic interactions in proteins and can affect the accuracy of predictions in computations [71]. It is controlled by the surrounding environment including the pH, temperature, and adjacent residues. In other words, the protonation and/or deprotonation will result in a different electrostatic charge on the side chain, which will strongly affect the FF parameters and promote and/or avert interactions. The H++ server (biophysics.cs.vt.edu) is used for prediction of protonation via estimation of pK constant values by study of continuum electrostatics and molecular mechanics [72]. The server generates ready-to-use files for MD simulations with predefined pH parameters. The most common protonation states are shown in Table 1. This tip is important for accuracy of molecular docking and conventional MD studies for applications of prediction of drug and substrate pose inside binding site. In such cases the protonation state is assumed only prior to the in silico experiment. To actually study the protonation and deprotonation transition in silico, numerous methods can be used which study the formation and breaking of covalent bonds, such as hybrid quantum mechanics and molecular mechanics (QM/MM) [73,74] or empirical valence bond (EVB) [75]. The applications of these methods are mostly evident in understanding enzyme–substrate interactions, substrate to product transition, and binding-site mechanisms. In QM/MM, the atoms are divided into a quantum mechanics system (simulating electrons and their interactions) and a molecular mechanics system. EVB does not require simulation of electrons and only applies the partial charges of atoms for calculations of energies of the valence bond resonance forms.

**Table 1:** The protonation states of polar and charged amino acids.

Amino acid	Charge	Three-letter code	Comment	Common choice for physiological pH
Arginine (ARG)	Positive	ARG	Protonated	Yes
	Neutral	ARN		
Aspartic acid (ASP)	Negative	ASP		Yes
	Neutral	ASH	Protonated	
Cysteine (CYS)	Neutral	CYS	Protonated	Yes
	Bridge	CYS or CYS2		In disulfide bridge
Glutamic acid (GLU)	Negative	GLU		Yes
	Neutral	GLH	Protonated	
Histidine (HIS)	Neutral	HIE	Protonated on Nε	Yes
	Neutral	HID	Protonated on Nδ	
	Positive	HIP	Protonated on Nδ and Nε	
Lysine (LYS)	Positive	LYS	Protonated	Yes
	Neutral	LYN		

## Tip 10: Understand the topologies you use

As mentioned earlier, the FF includes standard parameters (describing topologies) and equations involved in computations related to 3D structures. The application of FFs to estimate forces on atoms and bonds (represented by rigid spheres connected with springs) is called molecular mechanics. Many FFs have been developed over the years (Table 2). Ponder and Case wrote a historical narrative on the progress of FFs since 1980 to the present [76]. Early FFs did not explicitly use hydrogens in order to save computational costs (e.g., the united-atom FF), whereas the ones that explicitly use all the atoms are called all-atom FFs. Alternative types of FFs include the polarizable FFs, which were developed to face the limitations of using fixed partial atomic charges; however, they have limited applications to small structures. The parameters of molecular FFs, also known as topologies, are derived from both experimental and computational chemistry methods. For each FF, a different set of topologies are available depending on the different applications they were intended for. Some FFs either have or lack topologies for DNA, lipids, carbohydrates, or ions. Docking software often uses a general FF while MD software gives the user options to choose from. For MD, the earlier you learn how to read and edit topology files, the faster you can advance in the field and simulate complex molecules. Previous FFs use different atom types to describe various topologies (bond length; angle) of the same element. For example, oxygen in the general Amber FF (GAFF) [77] has three atom types: *o* (*sp*^*2*^ in carbonyl), *oh* (*sp*^*3*^ in hydroxyl), and *hs* (*sp*^*3*^ in ether and ester). A modern approach developed by the Open Force Field Consortium introduces a new definition of the FF by direct chemical perception, in contrast to traditional atom typing, by assigning the topologies directly based on substructures in the molecule [78].

**Table 2:** Common biomolecular FFs.

Force Fields	Example	Reference
AMBER	FF99SB: Primarily for proteins	[79]
CHARMM	C36: Proteins, nucleic acids, and lipids	[80]
GROMOS	54A7: Proteins and nucleic acids	[81]
OPLS	OPLS-AA: for organic molecules and peptides	[82]
Open FF	Smirnoff99Frosst: Open force field compatible to AMBER	[78]

AMBER, Assisted Model Building with Energy Refinement; CHARMM, Chemistry at Harvard Macromolecular Mechanics; FF, force field; GROMOS, GROningen Molecular Simulation; OPLS, Optimized Potentials for Liquid Simulations

Note that some programs like DeepView and Swiss-PdbViewer [23] use a united-atom FF (i.e., GROningen Molecular Simulation) GROMOS]). This means that when you are adding hydrogens (see Tip 4) there will be no explicit nonpolar hydrogens on the aliphatic carbons. In such cases, the user needs to remove hydrogens again before using some quality tools such as QMEAN server (described in Tip 8) [56].

## Modeling nonprotein molecules

The interactions between protein and nonprotein molecules can be studied by MD and other computational methods. While topologies of proteins, nucleic acids, lipids, and carbohydrates are feasible in some FFs, the case is more complicated for transition-metal ions, reactive substrates, and small drugs or ligands. All FFs have been optimized to best represent defined classes of molecules; therefore, introducing new “species” to the system requires careful attention. For the homology modeler, these nonprotein molecules differ in usefulness and complexity for the purpose of finding the native conformation in c-space or for identifying the protein function.

- **Water models**: Water plays important role in protein functions through hydrogen bonding. Fixed water molecules can be found in the ligand-binding site or on the interface between two interacting proteins and can influence the accuracy of molecular docking [71]. Explicit water models use molecules of 2 to 6 sites to represent water interactions. The most commonly used models in MD of proteins are rigid 3-sites (for the three atoms of H_2_O), such as simple point charge and TIP3P models, which can have modified topologies according to the FF. The small differences between the water models thus depend on the Van der Waals and electrostatic components [83]. Depending on the purpose of homology modeling, crystalized water molecules in the binding site for docking experiments can be retained (refer to Tip 3). For MD simulations, it is important to use a compatible water model with the FF.
- **Posttranslational modifications (PTMs):** The side-chain PTM has been largely ignored in the past until the Rosetta program started incorporating nonstandard amino acids in modeling. SIDEpro server (sidepro.proteomics.ics.uci.edu/) is one of the pipelines used for prediction of up to 15 of the most common PTMs [84]. PyTMs program also allows for introduction of 10 PTMs and basic structure optimization [85]. Identifying accessible residues for PTMs can also help in validation and refinement of loop modeling.
- **Noncovalent ligands**: For the purpose of docking, ligands are prepared by generating multiple conformations, and then a screening procedure is performed to select the top ranked conformer or ligand. In this case, the protein 3D structure has to be well optimized, or different sampling methods should be used to produce different structures of the native protein, e.g., by MD. The most common strategy among MD practitioners is to perform experiments with and without the ligand. Most MD programs can generate ligand topology based on general FF parameters, *e*.*g*. Antechamber [86] for AMBER and Chemistry at Harvard Macromolecular Mechanics (CHARMM) General Force Field for organic molecules (CGenFF) [87] for CHARMM. Other specialized servers and databases for preparing ready topology files include Automated for field Topology Builder (ATB) [88] and PRODRG [89] for both GROMACS and GROMOS. The generation of topology files, also known as parameterization, is done through quantum mechanics calculations. Study of ligands at the electron level provides more insight to their geometry, potential energy, and reactivity. These computations include the following: (1) Optimization of the ligand at a low level of theory (i.e., semiempirical molecular orbital level, e.g., AM1). Generally, this optimization requires fewer computational resources and can be sufficient for relatively large ligands (e.g., more than 50 atoms); (2) computation of the electrostatic potentials at deeper level of theory (e.g., ab initio level like Hartree–Fock molecular orbital or density functional theory level) for more accuracy [90]. The ultimate validation of the method can be achieved by comparing the findings with ligand’s NMR data. Notable ligand databases include PubChem (pubchem.ncbi.nlm.nih.gov/), Zinc (zinc.docking.org/) for commercially available ligands, Cambridge Structural Database (CSD, www.ccdc.cam.ac.uk/) for crystallographic data, and ChEMBL (www.ebi.ac.uk/chembl/) for bioactivity data.
- **Reactive substrates and covalent ligands**: The gold standard for study of reactions involving reactive substrates and covalent ligands is Hybrid QM/MM method, which can better describe the chemical bond breaking and formation, particularly in the study of enzyme and substrate reactions [91]. In contrast, classical MD alone cannot predict the formation or breaking of covalent bonds.
- **Metal ions**: Several approaches are under development for study of metal ions. In addition to the QM/MM method, it is possible to represent metal ions in classical MD simulations in either a nonbonded state only (comprising only electrostatic and Van der Waals topologies), a bonded state (holding bonds, angles, dihedrals, electrostatic, and Van der Waals topologies), or a dummy atom state, which usually constitutes a number of user-defined interaction sites.

## Case study

**Fig 2:**
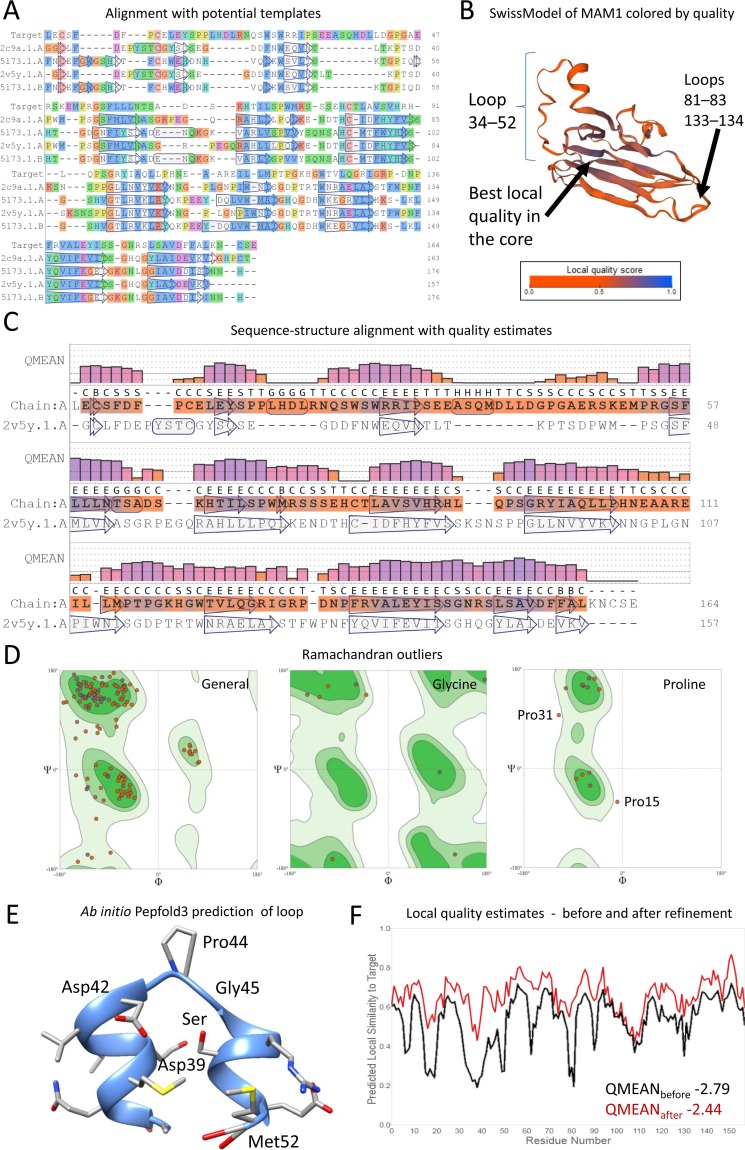
Case study: Homology modeling of the MAM1 domain in ALK receptor. (A) Alignment of MAM1 target sequence with potential templates. (B) Homology model built using SwissModel server using the 2v5y.1.A template 3D structure. QMEAN local estimates are shown in colored ribbon (worst in orange to best in blue). (C) Sequence–structure alignment of target template, showing secondary structures calculated via DSSP method and the quality QMEAN local scores in colored bars. (D) Ramachandran outliers for general, glycine, and proline residues. Representative Pro15 and Pro31 residues are shown. The Ramachandran plot shows the values of dihedral angles Φ and Ψ while green contours highlight the optimal expected values. (E) Ab initio prediction via the PEP-FOLD3 server for the region 35 to 52 (ASQMDLLDGPGAERSKEM) showing two α-helices with a GPGA linker. Since this region is relatively large and loop modeling often predicts loop secondary structures, we recommend using an ab initio approach as an additional source for homology modeling (as a template) for more accurate prediction. (F) Local QMEAN estimates before and after manual refinement. The major drops in quality before the refinements were corrected with selective optimization. The lowest QMEAN achieved was −2.44. At this stage, three remaining drops in regions 17 to 19, 37 to 40, and 106 to 112 were not correctable by optimization. ALK, anaplastic lymphoma kinase; DSSP, Dictionary of Secondary Structure of Proteins; QMEAN, Qualitative Model Energy Analysis.

In this case study, we shall build a homology model of a protein domain from one template using SwissModel server and refine it in the UCSF Chimera program [50] using quality information from the MolProbity and QMEAN servers. Anaplastic lymphoma kinase (ALK) is one of the most promising targets for treatment of neuroblastoma. Drug inhibitors of the intracellular ALK have been developed; however, little work has been done to target the extracellular part of ALK [92]. The MAM1 domain is one of two meprin/A5/mu (MAM) domains in the extracellular part of ALK receptor. MAM1 spans 264 to 427 residue range out of a total protein length of 1,620 residue (Uniprot ID: Q9UM73). For educational purposes, we will perform a basic homology modeling for MAM1 by selecting the best model in each round, whereas it is more common to work with several templates simultaneously for better results. Furthermore, we will perform manual refinement and quality checks. To begin, the sequence of our target is acquired from the Uniprot database and submitted in the SwissModel server platform to select the template. Note that the numbering (see Tip 4) has changed already, and we are dealing with 164 residues protein (numbered 1 to 164). For simplicity and educational purposes, we have kept the new numbering without change. The results show four 3D structures that cover most of the target sequence (Fig 2A). The templates (their range and coverage) were 2c9a.1.A (1-163/0.89), 2v5y.1.A (2-156/0.86), 5l73.1.A (1-162/0.88), and 5l73.1.B (1-162/0.88). This basic platform allows only for one-template modeling. Although other templates have better identities (with limited coverage), here we will only build models from these four templates for display. The templates with higher identity showed better QMEAN. Both 5l73 chains A and B with less than 20% identity failed the QMEAN test. The best model built was from 2v5y.1.A with a sequence identity of approximately 22%. It had a QMEAN score of −2.79, which is not very bad for such low identity (Fig 2B and 2C). By going to the structure assessment button, several panels show Ramachandran plots for outliers (for dihedral angles of general residues, glycine, proline, etc; Fig 2D), MolProbity analysis, and QMEAN plots.

At this point, we take three checklists: (1) For MolProbity backbone (side-chain outliers will be required for direct refinement), (2) for the low quality regions reported by QMEAN, and (3) for identifying the gaps in the alignment (see Tip 5), which are likely to correspond to the local quality estimates. The gaps were in 7 to 8, 16 to 22, 34 to 52, 64 to 67, 81 to 83, 92 to 93, 110 to 114, and 133 to 134. While the regions of few residues can be optimized, some other regions will require loop modeling performed later.

The model is saved in PDB format, and further refinement and optimization will be performed directly using graphical visualization software UCSF Chimera. Once the PDB file is opened, we add hydrogens (Tools>Structure Editing>AddH) and add partial charges (Tools>Structure Editing>Add Charge). For loop modeling (see Tip 6), we used local Modeller program via the UCSF Chimera to model the region 39 to 53 (Tools>Structure Editing>Model/Refine Loops). Out of 100 models, we were interested in a model that introduces a helix to that region. All peptides modeled from the region 35 to 52 (ASQMDLLD**GPGA**ERSKEM) using ab initio method via the PEP-FOLD3 server (bioserv.rpbs.univ-paris-diderot.fr/services/PEP-FOLD3/) show two helices separated by a flexible hinge 43 to 46 (GPGA) as featured in Fig 2E. Unfortunately, all loop models were stretched-loop shaped. In this unique situation where we speculate the actual model of the loop, the best decision would be to use this ab initio model as a second template for homology modeling. Otherwise, the modeler is advised to remove the regions that are difficult to model from the target sequence (e.g., use the same template sequence in that region at the alignment step if you do not want to have gaps in the chain). According to the authors who published this structure (see Tip 2), the MAM1 is glycosylated and contains two sodium ions. Interestingly, the loop is on the opposite side of the interaction interface (see Tip 1) [93].

Next, we start the optimization, refinement, and quality check. We selected the regions with low QMEAN quality for optimization (Favorites>Sequence, then use Ctrl+Shift keys to select residues with mouse). Here, we performed minimization as mentioned in Tip 7 (Tools>Structure Editing>Minimize Structure) using 3,000 steps SD then 1,000 steps CG on selected residues, whereas the remaining atoms were fixed. We do not recommend minimizing the whole structure during refinement and quality control because it can drastically affect the quality of the rest of residues. QMEAN and MolProbity servers can be used to recheck on the remaining residues requiring refinement (see Tip 8). Here, we found five residues with Ramachandran outliers (Pro15, Asp19, Pro31, Ala 46, and Pro53). The prolines can be directly fixed by changing their rotamers (Tools>Structure Editing>Rotamers) because the side chain involves their backbone, while the other amino acids can be selected one by one and optimized by selective minimization (100 steps SD or CG should be enough for each residue). Rotamer outliers were also changed using the same procedure described above. At some stage, the local QMEAN estimates will become difficult to fix once selective optimization has been overdone, and the remaining drops in quality will require additional loop modeling (Fig 2F). Once the selective refinement is finished, we remove the clashes (which were the main obstacle in our MolProbity score) by a final minimization of all structures (1,000 steps SD). The QMEAN score suffered a lot reaching −3.20 (due to averaging of lost peaks with previous drops in quality); however, MolProbity and clash scores are finally in the green zone at 1.81 and 1.62, respectively (initial MolProbity and clash scores were 2.45 and 10.93, respectively). Other elements, ions, and etc from the original template can be added by superposing the homology model and the crystal structure (Tools>Structure Comparison> MatchMaker).

## Conclusion

An English adage says, “A picture is worth a thousand words.” By the time you have ended your computational modeling study, it is important to accentuate on clarified presentation of protein 3D structures, as many renowned scientists (such as John Tukey [a statistician who developed modern graphical analysis] and Richard Feynman [a quantum physicist known for simplifying complex quantum concepts by the Feynman diagram]) have emphasized the benefits of simple graphical representation over tabulated data. We advise the modelers to present their results showing details with few distractions and with good annotations, taking into consideration the reader’s perspectives. The purpose of applying good modeling practices to produce high-resolution protein 3D models is to increase the level of accuracy of predictions. Computational study of protein 3D structures and their interactions with other molecules, their cellular functions, and mechanisms of action are among the leading scientific strategies for discovery. We hope that this educational article will help novices coming from different disciplines to follow practices that are far too costly to be learned by trial and error.
